# HMGB1 Accelerates Alveolar Epithelial Repair via an IL-1β- and αvβ6 Integrin-dependent Activation of TGF-β1

**DOI:** 10.1371/journal.pone.0063907

**Published:** 2013-05-16

**Authors:** Jean-François Pittet, Hidefumi Koh, Xiaohui Fang, Karen Iles, Sarah Christiaans, Naseem Anjun, Brant M. Wagener, Dae Won Park, Jaroslaw W. Zmijewski, Michael A. Matthay, Jérémie Roux

**Affiliations:** 1 Departments of Anesthesiology, University of Alabama at Birmingham, Birmingham, Alabama, United States of America; 2 Division of Pulmonary Medicine, Keio University School of Medicine, Tokyo, Japan; 3 Environmental Health Sciences, University of Alabama at Birmingham, Birmingham, Alabama, United States of America; 4 Department of Medicine, University of Alabama at Birmingham, Birmingham, Alabama, United States of America; 5 Division of Infectious Diseases, Korea University Ansan Hospital, Ansan, Republic of Korea; 6 Department of Medicine and Cardiovascular Research Institute, University of California San Francisco, San Francisco, California, United States of America; 7 Department of Systems Biology, Harvard Medical School, Boston, Massachusetts, United States of America; Università degli Studi di Milano, Italy

## Abstract

High mobility group box 1 (HMGB1) protein is a danger-signaling molecule, known to activate an inflammatory response via TLR4 and RAGE. HMGB1 can be either actively secreted or passively released from damaged alveolar epithelial cells. Previous studies have shown that IL-1β, a critical mediator acute lung injury in humans that is activated by HMGB1, enhances alveolar epithelial repair, although the mechanisms are not fully understood. Herein, we tested the hypothesis that HMGB1 released by wounded alveolar epithelial cells would increase primary rat and human alveolar type II cell monolayer wound repair via an IL-1β-dependent activation of TGF-β1. HMGB1 induced in primary cultures of rat alveolar epithelial cells results in the release of IL-1β that caused the activation of TGF-β1 via a p38 MAPK-, RhoA- and αvβ6 integrin-dependent mechanism. Furthermore, active TGF-β1 accelerated the wound closure of primary rat epithelial cell monolayers via a PI3 kinase α-dependent mechanism. In conclusion, this study demonstrates that HMGB1 released by wounded epithelial cell monolayers, accelerates wound closure in the distal lung epithelium via the IL-1β-mediated αvβ6-dependent activation of TGF-β1, and thus could play an important role in the resolution of acute lung injury by promoting repair of the injured alveolar epithelium.

## Introduction

Re-epithelialization of the distal lung during the recovery from acute respiratory distress syndrome (ARDS) is necessary to clear the edema fluid from the distal airspace of the lung and to restore a physiologic alveolar epithelial function [1]. In the distal lung, alveolar epithelial type II (ATII) cells have been shown to be a resident progenitor of alveolar epithelial regeneration [2], [3]. ATII cells re-establish alveolar epithelial barrier integrity by well-known mechanisms such as cell spreading and cell migration to cover the denuded area [2], [3]. To complete the restoration to normal morphological and functional properties of the alveolar epithelium, progenitor cells finally differentiate to alveolar type I and type II cells [4].

The initial loss of the epithelial barrier integrity is associated with the activation of a severe inflammatory response, resulting in increased numbers of neutrophils and increased concentrations of proinflammatory mediators including TNF-α, IL-1β, and TGF-β1, in the bronchoalveolar-lavage fluid (BALF) from patients with ALI [5]–[7]. Among these mediators, IL-1β was shown not only to increase lung vascular permeability, but also to enhance alveolar epithelial wound closure [2], [3]. In addition, we have shown in ATII cells that IL-1β activates TGF-β1, which in turn can increase alveolar epithelial wound closure [8], [9]. However, the prolonged presence of TGF-β1 in the alveolar space leads to pulmonary fibrosis [10]. The role of TGF-β1 in IL-1β-induced alveolar epithelial wound closure remains unknown.

High-mobility group box-1 (HMGB1) is a non-histone chromatin-associated protein that is actively secreted or passively released from necrotic or injured cells [11]. It is an important mediator of lung inflammation in experimental models of ALI from various origins (sepsis, trauma, ventilator-induced lung injury) [11]–[13]. Previous work has also reported that HMGB1 signals via Toll-like receptors (TLR-2, TLR-4, and the receptor for advanced glycation end-products RAGE to induce the nuclear translocation of NF-κB resulting in an enhanced production of proinflammatory cytokines, including TNF-α and IL-1β [14]–[16]. In contrast, HMGB1 inhibition attenuates lung inflammation in these preclinical models of ALI [11]–[13]. Finally, HMGB1 levels are increased in plasma and BALF of patients with ALI and correlate with outcome [11].

Extracellular functions of HMGB1 are not limited to inflammation. HMGB1 induces neuronal differentiation [17], and is a mitogen for vessel-associated stem cells [18] and for endothelial precursor cells [19]. Furthermore, HMGB1 promotes scratch wound closure of keratinocytes [20] and the topical application of HMGB1 corrects impaired would healing in diabetic skin [21]. However, the potential role of HMGB1 in stimulating alveolar epithelial wound closure has not been addressed. We hypothesized that HMGB1 is an early mediator of the alveolar epithelial wound closure. We found that HMGB1, released by primary rat ATII cell monolayers after scratch wound, enhanced the wound closure across primary cultures of rat and human alveolar epithelial cell monolayers via an IL-1β-dependent mechanism. Furthermore, we found that HMGB1 caused the release of IL-1β that resulted in a p38 MAP kinase-, RhoA- and αvβ6 integrin-dependent activation of TGF-β1 that enhanced epithelial alveolar wound closure by a PI3 kinase α-dependent mechanism.

## Materials and Methods

### Reagents

All cell culture media were prepared by the UCSF Cell Culture Facility using deionized water and analytical grade reagents. The PI3K inhibitors, PIK-90, PW12, TGX220 and SW14 were provided by Kevan M. Shokat (UCSF, San Francisco, CA) [22]. IC_50_ for each PI3K inhibitors are reported in **Table 1**. SB203580, an inhibitor of p38 MAP kinase was obtained from Calbiochem (San Diego, CA). Human recombinant TGF-β1 was obtained from R&D Systems (Minneapolis, MN). Antibodies and phosphoantibodies for Akt, p38 and MAP kinase dependent kinase were purchased from Calbiochem (San Diego, CA). Rabbit polyclonal anti– phospho and anti–total Smad2 was obtained from Cell Signaling Technology (Danvers, MA). Rat RAGE blocking antibody was obtained from R&D Systems (Minneapolis, MN). HMGB1 antibody and TLR4 blocking antibody were purchased from Abcam (Cambridge, MA). Goat anti-mouse and goat anti-rabbit IRDye®-conjugated secondary antibodies were purchased from LI-COR Biosciences (Lincoln, NE). Human recombinant HMGB1 purified in reducing conditions and provided in solution with DTT, IL-1β and IL-1β receptor antagonist (IL-1RA) were obtained from R&D Systems (Minneapolis, MN). Blocking studies were performed with anti-αvβ6 blocking (3G9) and type specific control antibodies (Ab), a generous gift from Gerald Horan (Biogen Idec, Cambridge, MA) [23]. TGF-β type II receptor (sTGFβRII) was a generous gift from Gerald Horan (Biogen Idec, Cambridge, MA), RhoA kinase (ROCK) inhibitor (Y-27632) was purchased from Calbiochem (San Diego, CA). ^125^I-labeled human serum albumin was purchased from Jeanatope (ISO-TEX Diagnostics, Friendswood, TX). Protein concentration of cell lysates was determined using the Bio-Rad protein assay kit (Bio-Rad, Hercules, CA). All other reagents were obtained from Sigma (St-Louis, MI).

**Table 1:** List of class I PI3K isoform inhibitors and their respective in vitro IC_50_.

Inhibitors	PI3Kα	PI3Kβ	PI3Kγ	PI3Kδ
PIK90	**11** **nM**	**350** **nM**	**18** **nM**	**58** **nM**
PW12	**15** **nM**	830 nM	970 nM	730 nM
SW14	8.9 µM	700 nM	**21** **nM**	**8.5** **nM**
TGX	784 nM	**5** **nM**	3.2 µM	**15** **nM**

Assays were conducted side by side with 10 µM ATP using the method described previously [22].

### Cell Culture

Primary cultures of rat and human alveolar epithelial cells were used for the *in vitro* studies. Rat alveolar epithelial type II (ATII) cells were isolated following approval from the University of California, San Francisco Institutional Animal Care and Use Committee (IACUC). Rat alveolar epithelial type II (ATII) cells were isolated as previously described [24], [25] with slight modifications. Briefly, cells were isolated by elastase digestion followed by negative selection using four monoclonal antibodies (mAbs) against cell surface molecules expressed on rat macrophages (CD4/CD32/CD45/RMA) purchased from BD Biosciences-Pharmingen (San Diego, CA). These mAbs were pre-incubated with Dynabeads M-450, magnetic beads with sheep anti-mouse IgG, (Dynal ASA, Oslo, Norway) in 0.1% BSA/PBS. After removing unbound mAbs, rat ATII cells were mixed with the bead suspension and rocked gently for 30 min at 4dC. Unbound cells were isolated and plated on polycarbonate Transwells (Corning Costar Co., Cambridge, MA) with a 0.4 µm pore size. Cells were seeded at a concentration of 1.5×10^6^ cells/cm^2^ in DMEM-H21 medium containing 10% low endotoxin fetal bovine serum, 1% penicillin and streptomycin and kept at 37°C in a humidified 95% air-5% CO_2_ incubator. Twenty-four hours later, nonadherent epithelial cells were removed by washing with PBS and fresh medium added to the lower compartments of the Transwells, thus maintaining the ATII cell monolayers with an air-liquid interface on their apical side. After 72–96 hours, cells that formed confluent monolayers reaching a transepithelial electrical resistance greater than 1500 ohms.cm^2^ were used for experiments.

Human alveolar epithelial type II cells were isolated from human lungs that were not used by the Northern California Transplant Donor Network following approval from the University of California, San Francisco Committee on Human Research. Our studies indicated that these lungs were in good condition, both physiologically and pathologically [26]. Cells were isolated after the lungs have been preserved for 4–8 hours at 4°C, using methods previously described [27]. A lobe of the human lung was selected that had no evidence of injury on the pre-harvest chest radiograph, could be normally inflated and had no area of consolidation or hemorrhage. The pulmonary artery for this segment was perfused with 37°C PBS solution and the distal airspaces of a segmental bronchus was lavaged 10 times with 37°C Ca2+, Mg2+ free PBS solution containing 0.5 mM EGTA and EDTA. Sixty to ninety ml of pancreatic porcine elastase (8 units/ml) diluted in a Ca2+, Mg2+free HBSS solution was instilled into the airspaces of 50 g of the chosen segment of lung tissue. The lung was incubated in a water bath for 30 min at 37°C and minced finely in the presence of fetal bovine serum and DNase I (500 µg/ml). The cell rich fraction was filtered sequentially through one-layer gauze, two-layer gauze, 150 µm and 30 µm nylon meshes. The cell suspension was then layered onto a discontinuous percoll density gradient 1.04–1.09 g/ml solution and centrifuged at 400×g for 20 min to remove red blood cells. The cells that accumulated at the interface of the solution and the percoll, were a mixture of type II pneumocytes and alveolar macrophages. These cells were recovered by centrifugation at 200×g for 10 min at 4°C. The pellet was resuspended in DMEM containing 10% FCS. The cells were incubated in DMEM containing magnetic beads coated with an anti-CD-14 antibody (Dynabeads M/450 CD14, Dynal, Oslo, Norway) at 4°C for 40 min under constant mixing to eliminate macrophages. The cell viability was assessed by trypan-blue exclusion. The purity of isolated human alveolar type II cells was checked by Papanicolaou staining or by staining with anti-human type II cell antibody (obtained from Leland Dobbs, UCSF) and the purity has consistently been more than 90%. Human alveolar type II cells were seeded on collagen I coated Transwells at a density of 1×10^6^ cells/cm2. The cells were grown in an air-liquid interface 72 h after seeding. Five days after the cells are seeded, the monolayer developed a transepithelial electrical resistance greater than 1500 ohms.cm^2^, as reported for rat ATII cell monolayers.

3T3 Fibroblasts were obtained from ATCC (Manassas, VA), and cultured in DMEM +10%FBS+P/S, according to ATCC standard protocols.

### Cell Viability

Cell viability after exposure to different experimental conditions was measured by the Alamar Blue assay [28]. Cell media were replaced by medium containing 10% Alamar Blue and placed at 37°C in the cell incubator for 2 to 3 hours. The media was then collected and read on a plate reader at 570 nm.

### S-Glutathionylation of HMGB1

HMGB1 glutathionylation was performed using previously described method [29], [30]. Purified HMGB1 (3 µg) in 100 µl PBS was incubated with GSH (0 or 100 µM) for 15 minutes, followed by exposure to H_2_O_2_ (200 µM) for an additional 15 minutes. Samples were then purified using spin desalting columns (Thermo Scientific) and amounts of non oxidized and oxidized HMGB1 determined using Western Blot analysis.

### Multiple Scratches Cell Supernatant (MS Cell Sup)

Primary rat ATII cells were grown to confluence in 6-well plates (1.5×10^6^ cells per well). Cells were washed three times with DMEM, and a sterile pipette was used to make 6 linear wounds. Cells were washed three times with DMEM to remove debris, and fresh DMEM (1 ml) was added to the monolayer for 6 hours before collection of the supernatant. Supernatant was directly used on rat ATII monolayers or kept frozen for later use. Condition media was fresh DMEM added to control cells (no scratch) for 6 hours before collection. MS Cell Sup in presence of 5 mM DTT caused a significant increase in epithelial wound closure, which was not statistically different than MS Cell Sup alone (data not shown).

### Wound Closure Analysis

Primary rat ATII cells were grown to confluence. Cells were washed three times with DMEM, and a sterile pipette was used to make a linear wound. Cells were washed three times with DMEM to remove debris. The degree of wound closure was quantified by calculating the percentage of the original wound area that was covered 16 h after wounding. Images were taken at 0 and 6 and 16 h (images were also taken at 24 h in preliminary studies). All experiments were done in triplicate. For each condition, three wounded areas were analyzed in parallel, and all experiments were repeated at least three times. Cell spreading, cell migration, and cell proliferation are well-known mechanisms of epithelial wound closure [2], [3]. To evaluate the contribution of proliferation on epithelial wound closure, we used mitomycin C (10 µg/ml), the DNA crosslinking inhibitor, in the cell media after wounding. We verified that mitomycin C treatment resulted in negligible BrdU incorporation in rat ATII cells, demonstrating an absence of DNA synthesis. Mitomycin C had no effect on MS Cell Sup, HMGB1, IL-1β, or TGF-β1-dependent increase in the degree of wound closure of primary rat ATII cell monolayers described in this study (data not shown).

### Depletion of HMGB1 by Immunoprecipitation

HMGB1 Ab (Novus Biologicals, Littleton, CO) was directly added (30 ug) to the MS Cell Sup obtained from 6-well plate monolayers (1 ml). HMGB1 was then immunoprecipitated using Protein A Dynabeads (Life technologies, Grand Island, NY) according to manufacturer’s instructions, and HMGB1-depleted supernatants were directly used in functional (wound closure) and biochemical assays (western blots).

### Bioassay for TGF-β Activation

Polarized rat ATII cells (5×10^5^ cells) were stimulated with IL-1β (10 ng/ml). Anti-αvβ6 (30 µg/ml) blocking Ab or their isotype control Ab were added 30 min before stimulation. Then, as we have described previously [31], mink lung epithelial reporter cells (TMLC; 5×10^4^ cells), expressing firefly luciferase under the control of the TGF-β sensitive plasminogen activator inhibitor-1 promoter, were seeded onto the ATII cell monolayer in absence or presence of blocking Ab. Co-culture was maintained for 16–20 h, and the final lysates were assayed for luciferase activity, as previously described [31].

### Measurement of Active TGF-β1

Rat active TGF-β1 levels were measured in triplicate by a commercially available enzyme-linked immunosorbent assay (ELISA) from R&D Systems (Minneapolis, MN), as we have done before [8]. The assay was performed according to the manufacturer’s protocol on primary rat alveolar type II cells. The sensitivity of the assay was 1.7 pg/mL active TGF-β1. This assay recognizes both natural and recombinant TGF-β1. No significant cross-reactivity or interference was observed.

### Measurement of IL-1β

IL-1β levels were measured in triplicate by a commercially available enzyme-linked immunosorbent assay (ELISA) from R&D Systems (Minneapolis, MN). The assays were performed according to the manufacturer’s protocol, in cell lysates. The sensitivity of the assay was less than 5 pg/ml. No significant cross-reactivity or interference was observed.

### Measurement of HMGB1

HMGB1 levels were measured in triplicate by a commercially available enzyme-linked immunosorbent assay (ELISA) from IBL International (Toronto, ON). The assays were performed according to the manufacturer’s protocol, in cell lysates. The sensitivity of the assay was less than 0.3 ng/ml. Cross-reactivity with HMGB2<2%.

### RhoA Activation Assay

RhoA activity was determined from endothelial and alveolar epithelial cells using the luminescence-based G-LISA™ RhoA activation assay biochemistry kit according to the manufacturer’s instructions (Cytoskeleton Inc., Denver, CO), as we have done before [8]. Briefly, cells were stimulated with IL-1β (10 ng/ml) for 10 min and lysed. The lysates were clarified by centrifugation at 4°C (8,000×g, 2 min), the protein concentration determined, and the final protein concentrations adjusted to 1.0 mg/ml. After incubating the lysates in the Rho-GTP affinity plate and adding the secondary Ab and detection reagents, luminescence was determined using the Wallac Victor 1420 (Perkin Elmer, Shelton, CT).

### Western Blot Analyses

Western blot analyses from frozen lungs and cells homogenates were performed as described previously [32]. After equal amounts of protein were loaded in each lane and separated by 10% SDS-PAGE, proteins were transferred to Invitrogen iBlot™ PVDF membranes (Invitrogen, Carlsbad, CA). Membranes were blocked for 1 hour with Odyssey blocking buffer (LI-COR Biosciences, Lincoln, NE), which was also used as primary and secondary antibodies incubation buffer. Primary antibodies were used at dilutions of 1∶500 and 1∶1000, incubated overnight at 4°C. Near-infrared (IR) detection was used with the IRDye®-conjugated secondary antibodies (LI-COR Biosciences, Lincoln, NE) which were either goat anti-mouse IRDye® 800CW or goat anti-rabbit IRDye® 680, used at 1∶10,000 dilution and imaged at 84 µm resolution with the Odyssey Infrared Imaging System (LI-COR Biosciences, Lincoln, NE). Quantification was performed with the LI-COR Biosciences analysis software.

#### Statistics

For the statistical analysis we used Statview Version 5.0 and MedCalc Version 7.2.0.2. The normal distribution was verified using the Kolmogorov-Smirnov test. For normally distributed data, data are summarized as means ± SEM. One-way analysis of variance and the Fisher’s exact t-test were used to compare experimental with control groups. For data that were not normally distributed, the data were subsequently analyzed with non-parametric tests and the data presented in the figures as box plots with medians, IQRs and lower and upper ranges. The Kruskal-Wallis test followed by the Dunn test was used to compare three or more experimental groups. The Mann-Whitney test was used to compare two experimental conditions. A *p* value of <0.05 was considered statistically significant.

## Results

### Endogenous HMGB1 Released by Damaged Primary Rat ATII Cells Increases Alveolar Scratch Wound Closure

**Figure 1:**
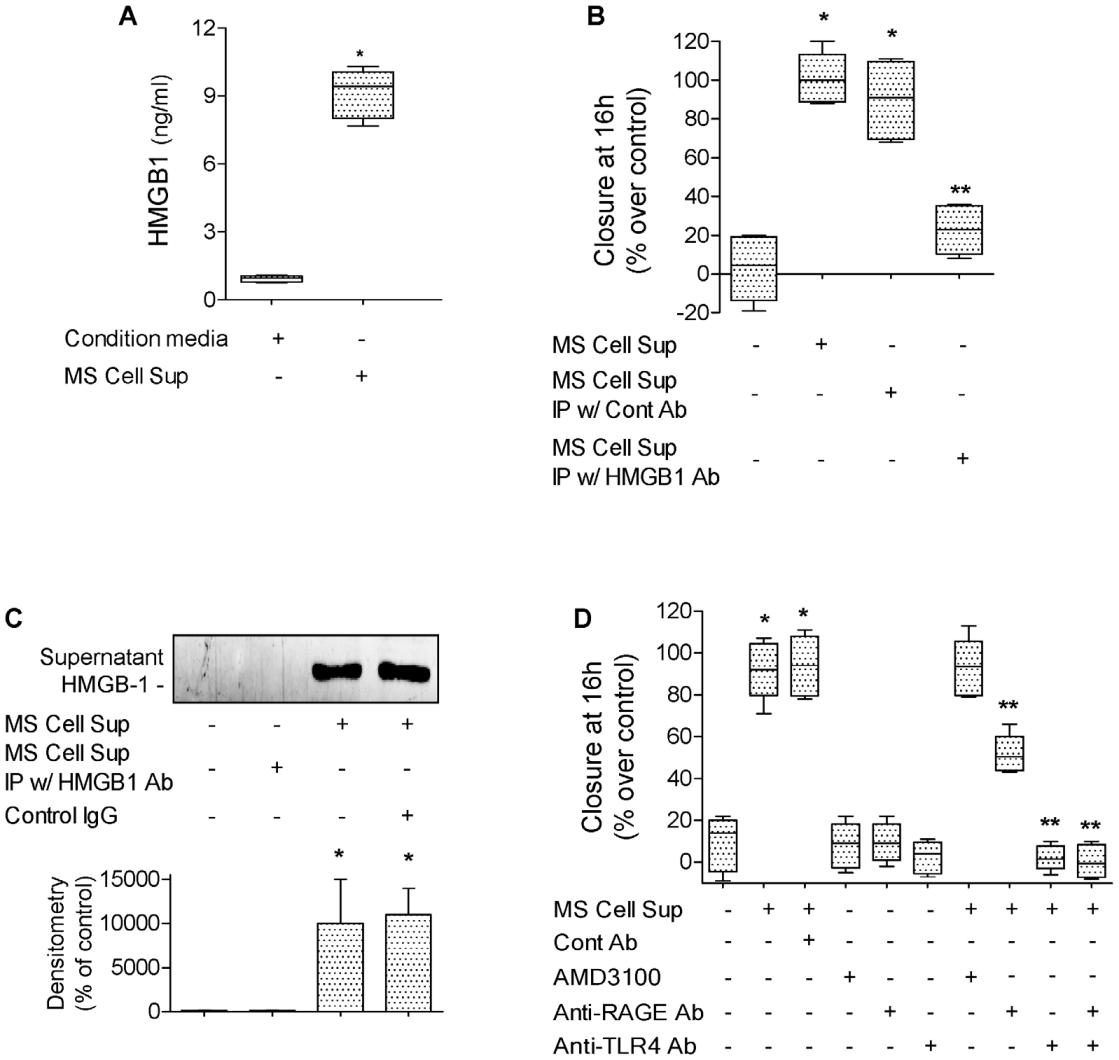
Endogenous HMGB1 released by damaged primary rat ATII cells increases alveolar epithelial wound closure. (**A**) HMGB1 was elevated in cell supernatant from rat ATII monolayers that underwent scratch wounds (MS Cell Sup) compared to cell supernatant from rat ATII monolayers that did not undergo scratch wounds (condition media). (**B**) MS Cell Sup increases the rate of wound closure of primary rat ATII cell monolayers compared to cell supernatant from rat ATII monolayers that did not undergo scratch wounds. HMGB1 was depleted from MS Cell Sup by immunoprecipitation using 30 µg/ml of HMGB1 specific Ab (MS Cell Sup IP w/HMGB1 Ab). Controls were MS Cell Sup immunoprecipitated with a control IgG (MS Cell Sup IP w/Cont Ab). (**C**) HMGB1 is secreted by primary rat ATII cell monolayers after scratch wounds. Multiple scratches (MS) were performed on primary rat ATII cell monolayers. Fresh cell media were added for 6 hours to the monolayers after extensive washes. Cell supernatants were then centrifuged to remove dead cells and cell debris, then analyzed by western blot (40 µl loaded per lanes from a 1 ml MS Cell Sup sample). (**D**) MS Cell Sup increases the rate of wound closure of a primary rat ATII cell monolayers via RAGE- and TLR4-dependent pathways, but not via a CXCR4-dependent mechanism. MS Cell Sup, and either 30 µg/ml of blocking RAGE or TLR4 antibodies or their isotype control IgG, or 1 µM of AMD3100, a CXCR4 inhibitor, were added to the monolayers after the scratch. Rate of wound closure is expressed as percent of control 16 h after wounding. *p<0.05 from monolayers exposed to control cell media; **p<0.05 from monolayers exposed to MS Cell Sup. For western blot experiments, one representative experiment is shown, three additional experiments gave comparable results; *p<0.05 from monolayers exposed to condition media.

HMGB1 is a multifunctional cytokine involved in inflammatory responses and in the repair of wounded skin. Therefore, we hypothesized that HMGB1, released during scratch wound of alveolar epithelial cell monolayers, could enhance alveolar epithelial wound closure. Collected supernatants from cell monolayers that underwent multiple scratch wounds (MS Cell Sup, see Methods) or from cell monolayers that did not undergo scratch wounds (condition media) were used in the following set of experiments. We found that HMGB1 was elevated in MS Cell Sup (9.1±0.9 ng/ml) compared to condition media of monolayers that did not undergo scratch wounds (0.9±0.1 ng/ml) measured by ELISA (**Figure 1A**). When added to primary rat ATII cell monolayers immediately after wounding, we found that MS Cell Sup caused a significant increase in epithelial wound closure, an effect not observed after depletion of HMGB1 by immunoprecipitation (**Figure 1B&C**). Since HMGB1 is known to signal through RAGE and TLR4 [14]–[16], and to induce migration by binding to CXCR4 when it forms a heterocomplex with CXCL12 [33], we added anti-RAGE, anti-TLR4 antibodies or the CXCR4 inhibitor AMD3100 with MS Cell Sup to primary rat ATII cell monolayers immediately after wounding. Although RAGE expression in ATII cells has been shown to increase overtime in culture [34], we found that pretreatment with an anti-RAGE antibody blocked 50% of the HMGB1 effect. Pretreatment with an anti-TLR4 antibody completely inhibited the MS Cell Sup-dependent increase in wound closure. Lastly, we found that the MS Cell Sup –dependent increase in wound closure is not CXCR4-dependent (**Figure 1D**).

### Endogenous HMGB1 Released by Damaged Primary Rat ATII Cells Increases Alveolar Scratch Wound Closure via an IL-1β-dependent Mechanism

**Figure 2:**
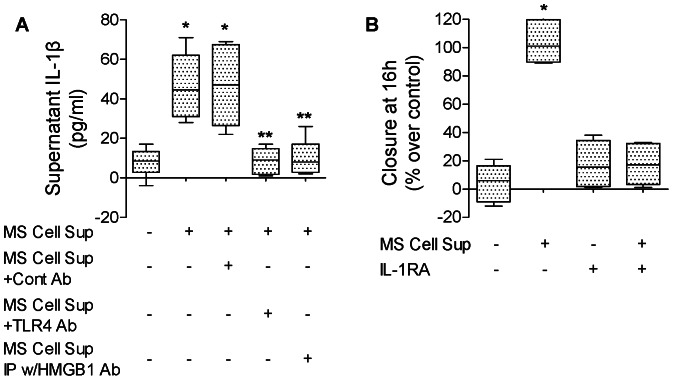
Endogenous HMGB1 released by primary rat ATII cell monolayers after scratch wounds increases alveolar epithelial wound closure via an IL-1β-dependent mechanism. (**A**) Supernatant from primary rat ATII monolayers collected 6 hours after multiple scratches (MS Cell Sup) induces an increase in the secretion of IL-1β by primary rat ATII cell monolayers via a TLR4-dependent pathway. MS Cell Sup, a blocking TLR4 antibody or its isotype control IgG were added to the monolayers after the scratch. HMGB1 was depleted from MS Cell Sup by immunoprecipitation using 30 µg/ml of HMGB1 specific Ab (MS Cell Sup IP w/HMGB1 Ab). Controls were MS Cell Sup immunoprecipitated with a control IgG (MS Cell Sup IP w/Cont Ab). IL-1β was measured by ELISA (see methods) in the cell supernatant. (**B**) IL-1β receptor antagonist (IL-1RA) prevented the MS Cell Sup-dependent increase in the rate of wound closure of primary rat ATII cell monolayers. MS Cell Sup, and IL-1β receptor antagonist (IL-1RA, 1 µg/ml) or its vehicle were added to the monolayers after the scratch. Rate of wound closure is expressed as percent of control 16 h after wounding. *p<0.05 from monolayers exposed to condition media. **p<0.05 from monolayers exposed to MS Cell Sup.

Since we have previously shown that IL-1β enhances *in vitro* alveolar epithelial wound closure [2] and because HMGB1 causes the release of IL-1β in lung endothelial cells [35], we next examined whether supernatants from cell monolayers that underwent multiple scratch wounds could increase the release of IL-1β by rat ATII cell monolayers. We found that MS Cell Sup increased the release of IL-1β by rat ATII cell monolayers, an effect that was blocked after depletion of HMGB1 by immunoprecipitation and by an antibody against TLR4, which is also a receptor for HMGB1 (**Figure 2A**). Then we tested whether the increase in wound closure due to MS Cell Sup was mediated through an IL-1β-dependent mechanism. We found that indeed IL-1RA prevented the increase in the rate of alveolar epithelial wound closure caused by MS Cell Sup (**Figure 2B**).

**Figure 3:**
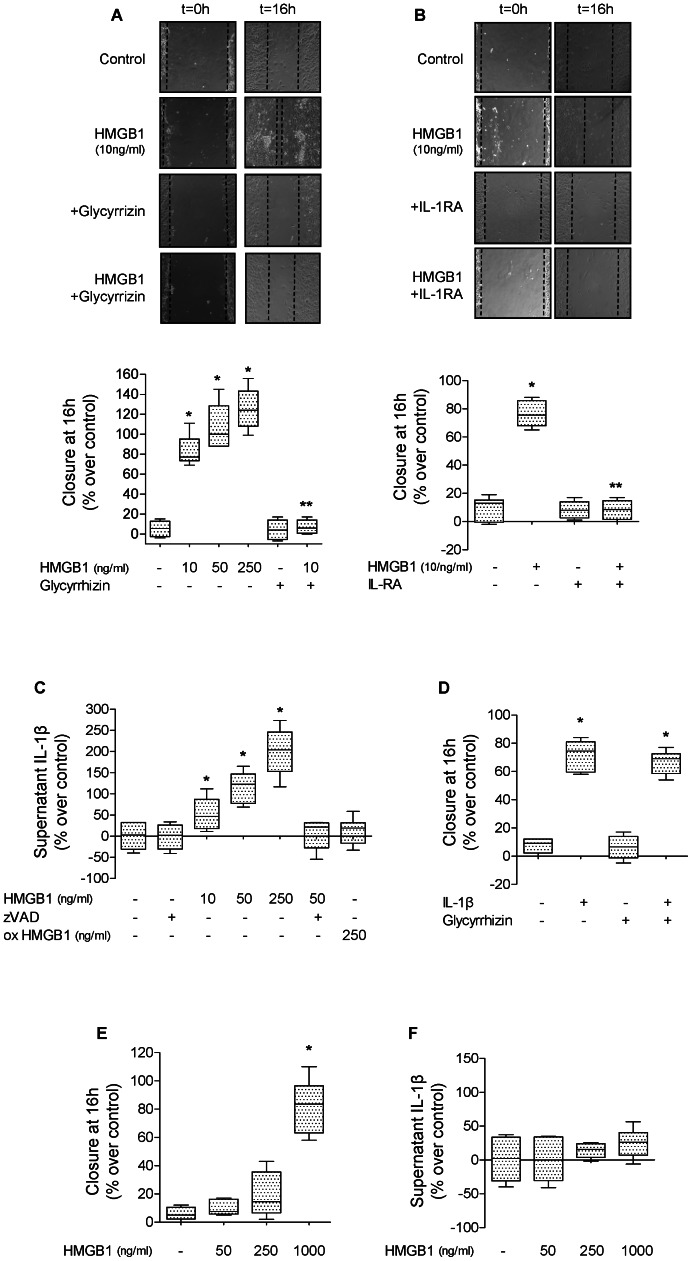
Human recombinant HMGB1 increases alveolar epithelial wound closure via an IL-1β-dependent signaling pathway. (**A**) Human recombinant HMGB1 increases the rate of wound closure of primary rat ATII cell monolayers. HMGB1 (10 ng/ml) or its vehicle was added to the monolayers after scratch. Phase contrast microscopy (20X magnification) immediately after wounding (left panels, t = 0 h) and after 16 h (right panels t = 16 h). Scale bar: 100 µm. In some experiments, glycyrrhizin or its vehicle was added to the monolayers after the scratch. (**B**) IL-1 receptor antagonist (IL-1RA) prevents HMGB1-dependent increase in rate of wound closure of primary rat ATII cell monolayers. HMGB1 (10 ng/ml) and/or IL-1 receptor antagonist (IL-1RA, 20 µg/ml) or their respective vehicles were added to the monolayers after the scratch. Phase contrast microscopy (20X magnification) immediately after wounding (left panels, t = 0 h) and after 16 h (right panels t = 16 h). Scale bar: 100 µm. (**C**) HMGB1 increases the secretion of IL-1β by primary rat ATII cell monolayers. HMGB1 (10, 50 and 250 ng/ml, 6 h) or fully oxidized HMGB1 (ox HMGB1, 250 ng/ml) or their respective vehicles was added to the cell monolayers, and IL-1β was measured by ELISA (see methods) in the cell supernatant. In some experiments, zVAD or its vehicle was added to the cell medium 30 min prior to HMGB1. (**D**) Glycyrrhizin prevents HMGB1-dependent increase in rate of wound closure of primary rat ATII cell monolayers. (**E**) High dose of Human recombinant HMGB1 (1 µg/ml) is required to increase the rate of wound closure of 3T3 fibroblasts. HMGB1 (50, 250, 1000 ng/ml) or its vehicle was added to the monolayers after scratch. (**F**) HMGB1 does not increase the secretion of IL-1β by 3T3 fibroblasts. HMGB1 (50, 250 and 1000 ng/ml, 6 h) or its vehicle was added to the cell monolayers, and IL-1β was measured by ELISA (see methods) in the cell supernatant. IL-1β (10 ng/ml) and glycyrrhizin (20 µg/ml) or their respective vehicles were added to the monolayers after the scratch. Rate of wound closure is expressed as percent of control 16 h after wounding. *p<0.05 from monolayers exposed to HMGB1 or IL-1β vehicles. **p<0,005 from monolayers exposed to HMGB1.

We next tested whether human reduced recombinant HMGB1 could reproduce the effect of endogenous HMGB1 released by wounded rat ATII cell monolayers. We found that human recombinant HMGB1 (10 ng/ml) increased by 80% the rate of wound closure, an effect that was blocked by glycyrrhizin, a specific inhibitor of HMGB1 or IL-1RA added to the cell monolayers immediately after wounding (**Figure 3A&B**). We then sought to determine whether HMGB1 would increase the secretion of IL-1β. We found that HMGB1 caused a dose-dependent release of IL-1β in ATII cell monolayers (control values: 8.3+1.1 pg/ml), an effect blocked by pretreatment with zVAD, an inhibitor of caspase-1, suggesting that HMGB1 induces the secretion of IL-1β via an activation of the NPLR3 inflammasome, which has been shown for lung endothelial cells [35] (**Figure 3C**). Although the fully reduced HMGB1 used in these experiments could perhaps be partially oxidized after been added to the cell medium of primary cultures of ATII cells, we tested the effect of a fully oxidized HMGB1 (see Methods) on the secretion of IL-1β required for the wound closure of ATII cell monolayers. The results show that oxidized HMGB1 does not induce the secretion of IL-1β by the ATII cell monolayers (**Figure 3C**). Finally, we found that IL-1β-dependent increase in the rate of alveolar epithelial wound closure was not affected by glycyrrhizin added to the cell monolayers immediately after wounding, indicating that IL-1β did not induce the release of HMGB1 by ATII cell monolayers and that IL-1β effect on the alveolar epithelial wound closure was HMGB1 independent (**Figure 3D**). Given the relatively high sensitivity of rat ATII cells to HMGB1, we performed additional control experiments in 3T3 fibroblasts. We found that 3T3 fibroblasts required a 100-fold higher concentration of HMGB1 to reach the same increase in the rate of wound closure over a 16 hour time period (**Figure 3E**). To better understand this cell-type specific difference, we also determined the amount of IL-1β secreted by 3T3 fibroblasts in response to exposure to HMGB1. We found that 3T3 fibroblasts showed no significant secretion of IL-1β even at very high doses of HMGB1 (1000 ng/ml) (**Figure 3F**). These results demonstrate a clear difference in sensitivity of ATII cells to HMGB1 when compared with 3T3 fibroblasts. Taken together, these results show that HMGB1 is released by primary rat ATII cell monolayers after wounding, and leads to a significant increase in alveolar epithelial wound closure via an IL-1β-dependent mechanism.

### IL-1β Increases Alveolar Scratch Wound Closure via a p38 MAP Kinase-, RhoA- αvβ6 Integrin- and TGF-β1-dependent Mechanism in Primary Rat and Human ATII Cell Monolayers

**Figure 4:**
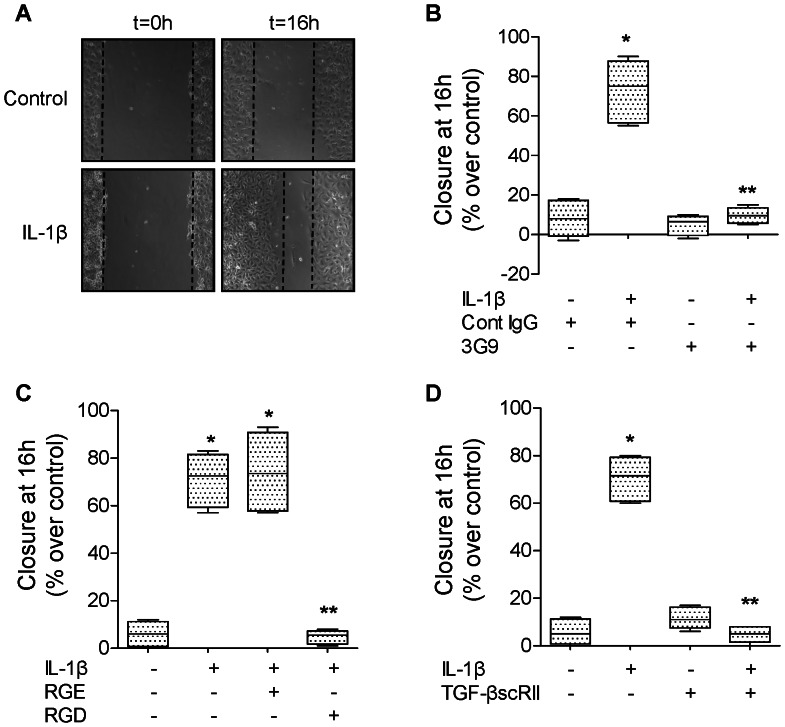
IL-1β increases wound closure via αvβ6 integrin and TGF-β1 in primary rat ATII cell monolayers. (**A**) IL-1β increases the rate of wound closure of primary rat ATII cell monolayers. IL-1β (10 ng/ml) or its vehicle was added to the monolayers after the scratch. Phase contrast microscopy (20X magnification) immediately after wounding (left panels, t = 0 h) and after 16 h (right panels t = 16 h). Scale bar: 100 µm. (**B**) A β6 blocking antibody (3G9) prevents IL-1β-dependent increase in rate of wound closure of a primary rat ATII cell monolayer. IL-1β (10 ng/ml). A β6 blocking antibody or its isotype control antibody was added to the monolayers after the scratch. (**C**) RGD peptides prevent IL-1β-dependent increase in rate of wound closure of a primary rat ATII cell monolayers. IL-1β (10 ng/ml) and RGE or RGD peptide were added to the monolayers after the scratch. (**D**) A TGF-β1 soluble receptor (TGF-βscRII) prevents IL-1β-dependent increase in rate of wound closure of primary rat ATII cell monolayers. IL-1β (10 ng/ml) and/or TGF-βscRII or their respective vehicles were added to the monolayers after the scratch. Degree of wound closure is expressed as percent of control 16 h after wounding. *p<0.05 from monolayers exposed to IL-1β vehicle. **p<0.05 from monolayers exposed to IL-1β.

**Figure 5:**
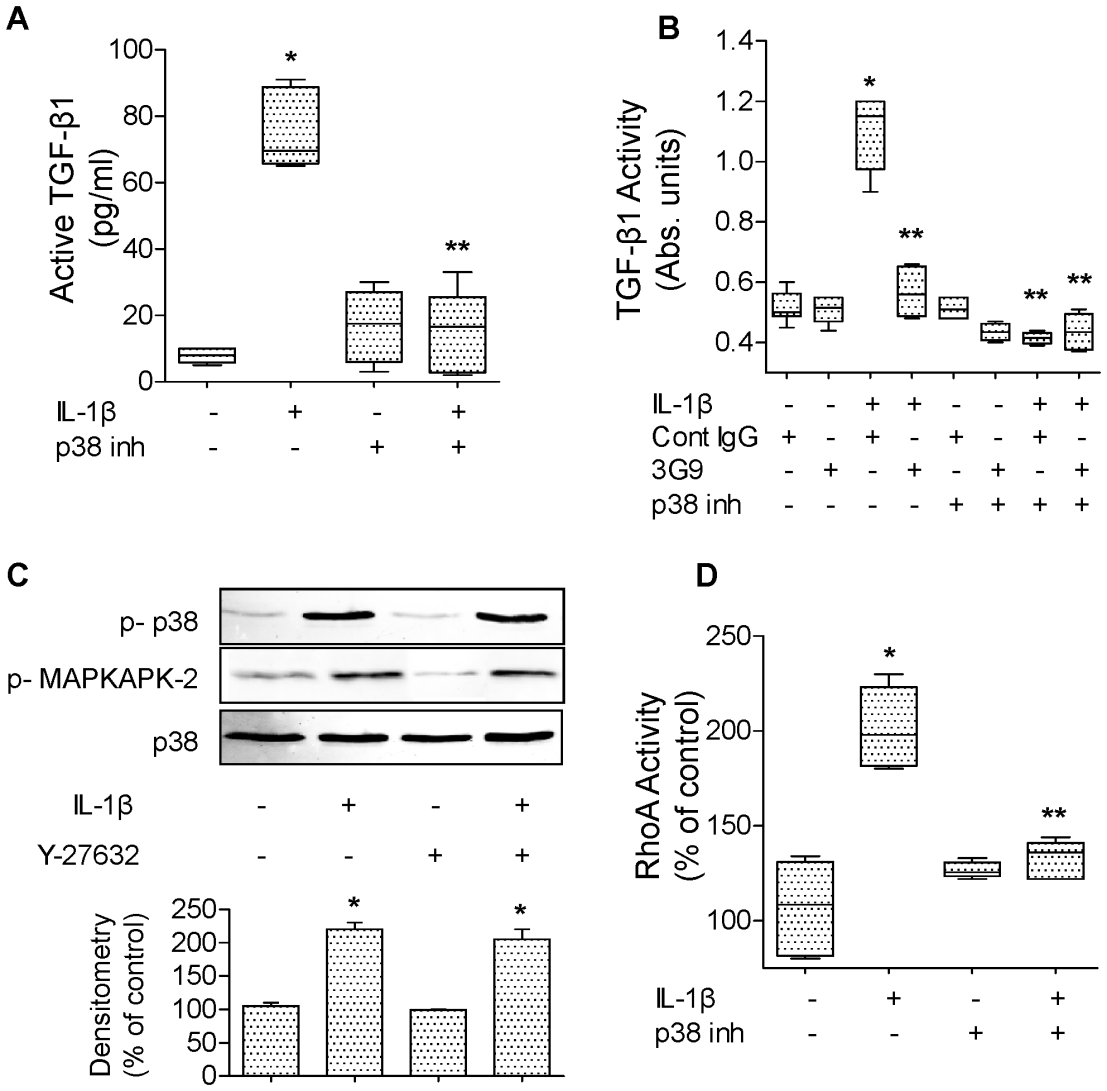
IL-1β induces a αvβ6 integrin-dependent activation of TGF-β1 via p38 and RhoA in primary rat ATII cell monolayers. (**A**) IL-1β increases active TGF-β1 expression via a p38 MAP kinase-dependent mechanism in primary rat ATII cell monolayers. IL-1β (10 ng/ml, 6 h) or its vehicle was added to the monolayers 30 minutes after pretreatment with a p38 MAP kinase inhibitor (SB202190, 10 µM) or its vehicle. Active TGF-β1 was measured by ELISA, as described in the methods. (**B**) IL-1β increases TGF-β1 activity via a β6 integrin and p38 MAP kinase-dependent mechanism in primary rat ATII cell monolayers. IL-1β (10 ng/ml, 6 h) or its vehicle was added to the monolayers, 30 minutes after pretreatment with either a β6 blocking antibody, an isotype control antibody, a p38 MAP kinase inhibitor (SB202190, 10 µM) or its vehicle. Active TGF-β1 was measured using mink lung epithelial reporter cells (TMLC, Bioassay for TGF-β1 as described in the methods). (**C**) RhoA inhibition does not prevent p38 MAP kinase activation by IL-1β in primary rat ATII cell monolayers. IL-1β (10 ng/ml, 10 min) or its vehicle was added to the monolayers 30 minutes after pretreatment with RhoA inhibitor (Y-27632, 10 µM) or its vehicle. (**D**) A p38 MAP kinase inhibitor (SB202190) prevents RhoA activation by IL-1β in primary rat ATII cell monolayers. IL-1β (10 ng/ml, 30 min) or its vehicle was added to the monolayers, 30 minutes after pretreatment with a p38 MAP kinase inhibitor (SB202190, 10 µM) or its vehicle. *p<0.05 from monolayers exposed to IL-1β vehicle. **p<0.05 from monolayers exposed to IL-1β. For western blot experiments, one representative experiment is shown, three additional experiments gave comparable results; *p<0.05 from monolayers exposed to IL-1β vehicle.

**Figure 6:**
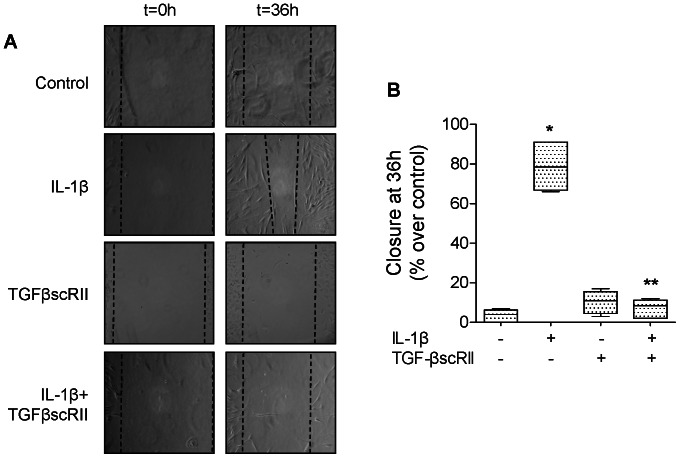
IL-1β increases wound closure via a TGF-β1-dependent mechanism in primary human ATII cell monolayers. (**A**) IL-1β increases the rate of wound closure in primary human ATII cell monolayers. IL-1β (10 ng/ml) and/or TGF-βscRII or their respective vehicles were added to the monolayers after the scratch. Phase contrast microscopy (20X magnification) immediately after wounding (left panels, t = 0 h) and after 36 h (right panels t = 36 h). Scale bar: 100 µm. (**B**) TGF-β1 soluble receptor (TGF-βscRII) prevents IL-1β-dependent increase in rate of wound closure of primary human ATII cell monolayers. IL-1β (10 ng/ml) and/or TGF-βscRII or their respective vehicles were added to the monolayers after the scratch. Rate of wound closure was expressed as percent of control 16 h after wounding. *p<0.05 from monolayers exposed to IL-1β vehicle. **p<0.05 from monolayers exposed to IL-1β.

IL-1β (10 ng/ml, 16 hours) significantly increases the rate of wound closure in primary rat ATII cell monolayers (**Figure 4A**) via an αvβ6 integrin- and TGF-β1-dependent mechanism. The IL-1β effect was also blocked by treating the cell monolayers immediately after wounding with either a blocking antibody against αvβ6 integrin, a RGD peptide or a TGF-β1 soluble chimeric receptor II (TGF-βscRII) (**Figure 4B–D**). We next examined the signaling pathways implicated in the activation of TGF-β1 via the αvβ6 integrin. We found that inhibiting the activation of p38 MAPK prevented the IL-1β-dependent activation of TGF-β1 (**Figure 5A**). Using mink lung epithelial reporter cells expressing luciferase under the control of the TGF-β sensitive plasminogen activator inhibitor-1 promoter, we showed that blocking αvβ6 integrin did not have any additive effect to p38 MAPK inhibition on the IL-1β-dependent activation of TGF-β1 (**Figure 5B**). Furthermore, inhibiting RhoA by an inhibitor of the RhoA-dependent kinase (Y27632) did not affect the IL-1β-mediated activation of p38 MAP kinase nor the phosphorylation of its downstream kinase MAPKAPK-2 (**Figure 5C**). In contrast, we found that inhibiting p38 MAP kinase prevented RhoA activation by IL-1β (**Figure 5D**), indicating that RhoA activation by IL-1β is downstream of the activation of p38 MAP kinase. Finally, to further validate the previous observations in rat ATII cell monolayers, we also demonstrated that IL-1β increased alveolar epithelial wound closure via a TGF-β1-dependent mechanism in primary human ATII cells (**Figure 6A&B**). Taken together, these experiments demonstrated that the IL-1β-enhanced alveolar scratch wound closure via an αvβ6 integrin-dependent activation of TGF-β1 in ATII cell monolayers.

### TGF-β1 Increases Alveolar Scratch Wound Closure via PI3Kα-dependent Mechanism

**Figure 7:**
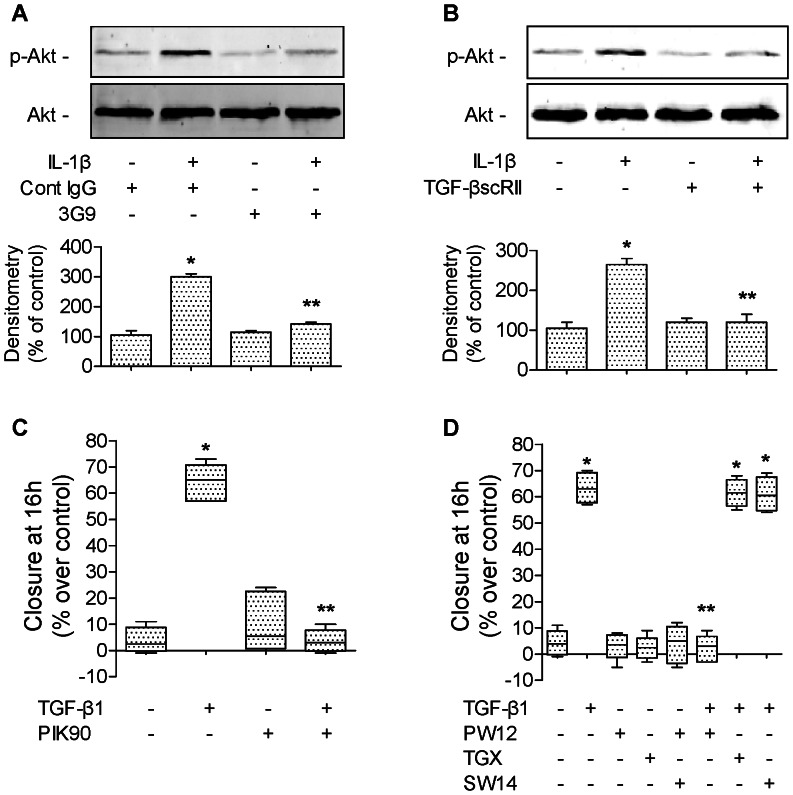
TGF-β1 increases alveolar epithelial repair via PI3Kα in primary rat ATII cell monolayer. (**A**) A β6 blocking antibody (3G9) prevents IL-1β-dependent phosphorylation of Akt in primary rat ATII cell monolayers. IL-1β (10 ng/ml, 30 min) or its vehicle was added to the monolayers 30 minutes after pretreatment with a β6 blocking antibodies or its isotype control antibody (3G9, Cont IgG, 1 µg/ml). (**B**) TGF-β1 soluble receptor (TGF-βscRII) prevents IL-1β-dependent phosphorylation of Akt in primary rat ATII cell monolayers. IL-1β (10 ng/ml, 30 min) or its vehicle was added to the monolayers 30 minutes after pretreatment with a TGF-β1 soluble receptor (TGF-βscRII, 20 µg/ml) or its vehicle. (**C**) PI3 kinase inhibition prevents TGF-β1-dependent increase in rate of wound closure of primary rat ATII cell monolayers. TGF-β1 (10 ng/ml) and a broad inhibitor of PI3K (PIK90, 1 µM) or their respective vehicles were added to the monolayers after the scratch. (**D**) Inhibition of PI3Kα prevents TGF-β1-dependent increase in rate of wound closure of primary rat ATII cell monolayers. TGF-β1 (10 ng/ml) and isoform-specific inhibitors of PI3K (PW12, TGX220, SW14, 0.5 µM) or their respective vehicles were added to the monolayers after the scratch. See **Table 1** for PI3K isoform-specific inhibitors IC_50_. Rate of wound closure is expressed as percent of control 16 h after wounding. *p<0.05 from monolayers exposed to TGF-β1 vehicle. For western blot experiments, one representative experiment is shown, three additional experiments gave comparable results; *p<0.05 from monolayers exposed to IL-1β vehicle. For immunofluorescence experiments, one representative experiment is shown; four additional experiments gave comparable results.

Because PI3 kinase has been implicated in epithelial cell migration [36], we determined if PI3 kinase signaling mediates the IL-1β- and TGF-β1-dependent increase in alveolar epithelial wound closure. We found that IL-1β increased Akt phosphorylation on its serine 473 in rat ATII cells (**Figure 7A**). The IL-1β-induced Akt phosphorylation was blocked by both a αvβ6 integrin blocking antibody (**Figure 7A**) and a TGF-β1 soluble chimeric receptor II (TGF-βscRII) (**Figure 7B**). These results indicate that TGF-β1 mediates the IL-1β-dependent activation of PI3 kinase through the αvβ6 integrin. Secondly, we found that PI3 kinase inhibition completely blocked the TGF-β1-dependent increase in alveolar epithelial wound closure (**Figure 7C**). Finally, using isoform-specific PI3 kinase inhibitors, we found that pretreatment with an inhibitor of PI3Kα (PW12), but not of PI3Kβ, γ and δ, prevented the IL-1β-induced increase in epithelial wound closure (**Figure 7D**). Taken together, these data indicate that the IL-1β-mediated increase in epithelial wound closure in primary rat ATII cell monolayers is mediated via a p38 and RhoA -dependent activation of TGF-β1 by the integrin αvβ6 and active TGF-β1 triggers an increase in wound closure via a PI3Kα-dependent mechanism.

### Endogenous HMGB1 Released by Wounded Primary Rat ATII Cell Monolayers Increases Alveolar Scratch Wound Closure via an IL-1β and αvβ6 Integrin -dependent Activation of TGF-β1

**Figure 8:**
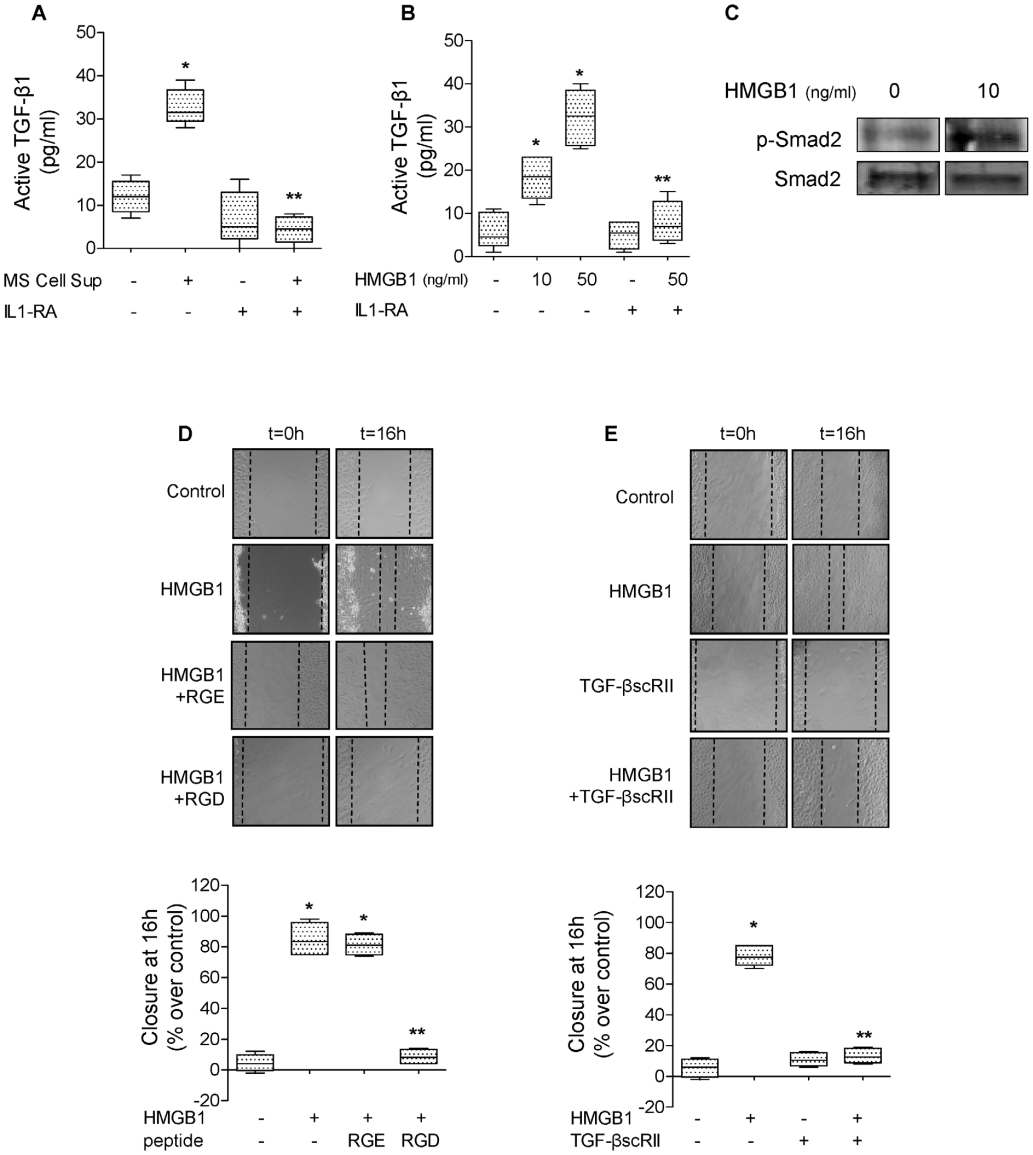
HMGB1 augments alveolar epithelial wound closure via TGF-β and IL-1β in primary rat ATII cells. (**A**) Supernatant from primary rat ATII monolayers collected 6 hours after multiple scratches (MS Cell Sup) induces the activation of TGF-β1, which is t blocked by IL-1 receptor antagonist (IL-1RA). MS Cell Sup, and IL-1RA (1 µg/ml) or its vehicle were added to the monolayers for 6 hours. Active TGF-β1 was measured by ELISA, as described in the methods. (**B**) IL-1 receptor antagonist (IL-1RA) prevents HMGB1-dependent activation of TGF-β1 in primary rat ATII cell monolayers. HGMB1 (10 and 50 ng/ml, 6 h) or its vehicle was added to the monolayers 30 min after pretreatment with IL-1RA or its vehicle. (**C**) Human recombinant HMGB1 (10 ng/ml, 1 h) causes Smad2/3 phosphorylation in rat primary ATII cell monolayers. (**D**) RGD peptides prevent HMGB1-dependent increase in rate of wound closure of primary rat ATII cell monolayers. HMGB1 (10 ng/ml) or its vehicle and RGE or RGD peptides (20 µg/ml) were added to the monolayer after the scratch. Phase contrast microscopy (20X magnification) immediately after wounding (left panels, t = 0 h) and after 16 h (right panels t = 16 h). Scale bar: 100 µm. (**E**) A TGF-β1 soluble receptor (TGF-βscRII) prevents HMGB1-dependent increase in the rate of wound closure of primary rat ATII cell monolayers. HMGB1 (10 ng/ml) and TGF-β1 soluble receptors (TGF-βscRII, 20 µg/ml) or their respective vehicles were added to the monolayers after the scratch. Phase contrast microscopy (20X magnification) immediately after wounding (left panels, t = 0 h) and after 16 h (right panels t = 16 h). Scale bar: 100 µm. Active TGF-β1 was measured by ELISA, as described in the methods. Rate of wound closure is expressed as percent of control 16 h after wounding. *p<0.05 from monolayers exposed to control cell media or HMGB1 vehicle. **p<0.05 from monolayers exposed to MS Cell Sup or HMGB1.

In the last series of experiments, we tested the hypothesis that supernatants from wounded cell monolayers (MS Cell Sup) and human recombinant HMGB1 were both sufficient to activate TGF-β1 via an IL-1β signaling pathway in ATII cell monolayers. We found that MS Cell Sup activated TGF-β1, an effect that was blocked by IL-1 receptor antagonist (IL-1RA) (**Figure 8A**). Furthermore, human recombinant HMGB1 (10 and 50 ng/ml) also activated TGF-β1, an effect also blocked by IL-1RA (**Figure 8B**). We further confirmed these results by performing a western blot analysis that showed that HMGB1 also caused phosphorylation of Smad2/3 in ATII cells (**Figure 8C**). Finally, we demonstrated that a RGD blocking peptide or TGF-βscRII added immediately after wounding inhibited the HMGB1-induced increase in the rate of wound closure of rat ATII cell monolayers (**Figure 8C&D**). Taken together, these data demonstrate that HMGB1, released by wounded ATII cell monolayers, activates TGF-β1 via an IL-1β- and αvβ6-dependent mechanism, thus enhancing wound closure in primary rat ATII cell monolayers.

## Discussion

Acute lung injury (ALI) is a clinical syndrome manifested by the rapid onset of respiratory failure associated with high mortality [37]. ALI is characterized by increased permeability of the alveolar-capillary barrier, decreased surfactant function, and impaired alveolar fluid clearance [1]. Importantly, a small subset of patients with ALI who do not have severe epithelial damage and thus retain maximal alveolar fluid clearance have better clinical outcomes [3]. For the patients whose alveolar epithelium is injured, the re-epithelialization of the distal lung is crucial to clear the edema fluid and restore normal lung function. The mechanisms implicated in the repair of the alveolar epithelium are complex and include spreading, migration, proliferation and differentiation of resident progenitor cells (such as ATII cells) and local or bone marrow-derived stem cells [38]. Although the definitive identification of alveolar epithelial progenitor cells remains elusive, recent work has suggested that there is a stable progenitor population of alveolar epithelial cells in the lung that are positive for the integrin α6β4 and can respond dynamically to injury by proliferation and differentiation [39].

Alveolar epithelial repair can be stimulated by multiple factors including soluble ligands, structural elements, signaling pathways and mechanical environment [40]. During the last decade, there is been a growing body of evidence that growth factors and inflammatory mediators released in the alveolar milieu during the acute phase of ALI not only cause damage to lung epithelial cells, but may also accelerate physiologic alveolar epithelial repair or induce a dysfunctional repair that results in the development of lung fibrosis. Importantly, damaged ATII cell monolayers can repair themselves in the absence of exogenous factors suggesting that autocrine modification of the ATII cell monolayer milieu promotes alveolar epithelial repair. For example, recent work has shown that soluble autocrine factors, such as CINC-1 and ICAM secreted by damaged ATII cell monolayers in response to cell damage, play a functional role in alveolar epithelial healing process [38]. HMGB1, a member of the damage associated molecular patterns family (DAMPs) that is actively secreted or passively released from necrotic or injured cells [11], has been shown to be an important inflammatory mediator in several experimental models of acute lung injury [11]–[13] and has been detected in the alveolar milieu of patients with lung injury [41], [42]. However, HMGB1 has also been shown to promote scratch wound closure of keratinocytes [20] and to correct impaired would healing in diabetic skin [21], although its potential role in stimulating alveolar epithelial wound closure has not been addressed. In the present study, we found that HMGB1, released by primary rat ATII cell monolayers after scratch wound, enhanced the rate of wound closure when added to primary rat ATII cell monolayers immediately after wounding. The concentration of endogenous HMGB1 released in the extracellular space by damaged ATII cells was sufficient to stimulate alveolar scratch wound closure, an effect that was lost when endogenous HMGB1 was removed from the cell supernatant prior to exposure to wounded ATII cell monolayers. This result indicates that in addition to its role as endogenous inflammatory mediator, HMGB1 may play an important role in the repair of the lung epithelium after severe injury.

What are the signaling pathways by which HMGB1 stimulates alveolar epithelial wound closure? Multiple studies have previously reported that HMGB1 signals via toll-like receptors, RAGE and CXCR4; whether HMGB1 stimulate these receptors on alveolar epithelial cells is still unknown. Previous *in vitro* work has shown that HMGB1 acting through TLR4 and a synergistic collaboration with TLR2 and RAGE signaling, mediates inflammasome activation and IL-1β secretion from lung endothelial cells [35]. Another study reported that HMGB1 increases lung endothelial permeability via RAGE as the primary receptor signaling the HMGB1-induced increase in paracellular permeability and intercellular gap formation [16]. Furthermore, HMGB1 also modulates the response of immune cells in acute lung injury. For example, HMGB1 has been shown to inhibit phagocytosis of apoptotic neutrophils by macrophages *in vivo* and *in vitro*. Phosphatidylserine was directly involved in the inhibition of phagocytosis by HMGB1, as blockade of HMGB1 by this mediator eliminates the effects of HMGB1 on efferocytosis [43]. Finally, *in vivo* work demonstrated that HMGB1 contributes to the development of lung injury after severe hemorrhage [12] and that HMGB1-mediated lung inflammation depends on TLR4 in the early phase and on TLR2 in the late phase following hemorrhagic shock [41]. However, despite this body of experimental work, it was still not known if HMGB1 stimulates these receptors on alveolar epithelial cells. A recent study by Schiraldi *et al.* [33], has shown that CXCR4 is important for cell motility in response to HMGB1 in fibroblasts and monocytes. These authors demonstrated that elevated concentrations of HMGB1/CXCL12 heterocomplexes induced cell migration by binding to CXCR4 in these cell types. However, using the CXCR4 inhibitor AMD3100, we found that the effect of HMGB1 wound closure was not mediated through CXCR4 in primary rat ATII cells. In contrast, the results of the present study indicate that blocking TLR4 completely inhibited the effect of HMGB1, while blocking RAGE only inhibits 50% of the HMGB1 effect on alveolar scratch wound closure, indicating that these two receptors play an important role in HMGB1 signaling in the alveolar epithelium. Although several studies have reported the potential existence of cross-talk between these receptors [44], further examinations would be required to determine its importance for the observations presented here.

The third important finding of the present study is that HMGB1 mediates its effect via an IL-1β-αvβ6 integrin-dependent activation of TGFβ1. We have previously reported that IL-1β can activate the αvβ6 integrin and TGFβ1 in alveolar epithelial cells that results in an increase in paracellular epithelial permeability [8]. Other investigators have reported that thrombin, PAR-1 agonists, and lysophosphatidic acid (LPA) via its receptor 2 are also able to activate this pathway [45]–[47]. We found in the present study that the extracellular release of IL-1β by HMGB1 is inhibited by a caspase-1 inhibition suggesting the involvement of the NPLR3 inflammasome, as it has previously been shown in lung endothelial cells [35]. However, it should be point out that HMGB1 can also form protein complexes with cytokines or TLR-ligands to enhance inflammatory mediator release [48]–[50]. Furthermore HMGB1, via the release of IL-1β, activates the αvβ6 integrin by causing a contraction of the epithelial cells via a p38 MAP kinase and RhoA-dependent mechanism. This result is in accordance with a previous work that has shown that LPA and PAR-1 agonists activate RhoA via GαQ, but not Gαi or Gα12/13, and cause cytoskeletal reorganization leading to epithelial contraction and activation of the αvβ6 integrin and TGFβ1 [45]. However, there are other mechanisms by which TGFβ1 can be activated such as physical processes, including acidification, temperature changes and oxidation, proteases including plasmin, tryptase, elastase and matrix metalloproteinases (MMP)-2 and 9 and interaction with thrombospondin (reviewed in [51]). Interestingly, HMGB1 has been shown to activate MMP2 and 9 in in human lung epithelial cancer cells [40]. Furthermore, the αvβ6 integrin can upregulate MMP9 and promotes migration of normal oral keratinocytes [3]. Thus, as our results indicate that in the present *in vitro* model the inhibition of the αvβ6 integrin with blocking antibody or small peptides completely prevented the effect of HMGB1 and IL-1β on alveolar epithelial wound closure. Our data do not exclude that there is a potential role for MMP9 in directly activating TGF-β1 by cleavage from the latent associated protein in addition to the known spatially restricted activation of this mediator by the αvβ6 integrin.

Is TGF-β1 implicated in the process of alveolar epithelial wound closure? Previous studies have provided evidence that TGF-β1 accelerates the wound closure in primary alveolar type II and bronchial epithelial cells [9], [52], [53], although its activation by another integrin, αvβ8, delays bronchial epithelial wound closure [54]. We demonstrated here that the effect of TGF-β1 is mediated by its activation of the PI3Kα. These results are in accordance with those of Rogel et al. that reported a critical role for vimentin in the wound closure of alveolar epithelial cell monolayers mediated by TGF-β1 [53]. Indeed, a recent report shows that TGF-β1 increases the expression of vimentin via PI3K/Akt- and MAP kinase ERK1/2-dependent mechanisms in A549 cells, an alveolar epithelial cell line [55]. In addition to TGF-β1 enhancing alveolar epithelial wound closure, it plays an important role in the development of lung fibrosis during the late phase of acute lung injury via epithelial-mesenchymal transformation (EMT) of alveolar epithelial cells [56]. Recent experimental work has suggested new mechanisms to explain some of the prior observations that TGF-β1 can have opposing effects depending on the response of the epithelial cell to injury. Repeated injury associated with persistent inflammation and hypoxia may overwhelm normal lung repair mechanisms and create sustained fibrogenesis [56]. One recently discovered mechanism is related to the disruption of cell contacts with the basement membrane via the α3β1 integrin and laminin followed by the formation of complexes between the α3β1 integrin, E-cadherin, β-catenin, and TGF-β1 receptors. These protein complexes cause the phosphorylation of β-catenin and Smad-2 and drive EMT [56].

In summary, this study demonstrates that the HMGB1 released by wounded epithelial cell monolayers, accelerates wound closure in the distal lung epithelium via the IL-1β-mediated αvβ6-dependent activation of TGF-β1, and thus could play an important role in the resolution of acute lung injury by promoting repair of the injured alveolar epithelium. These results also indicate that after injury a limited lung inflammatory response may have a beneficial effect on the tissue repair and restoration of the organ function, suggesting that a complete inhibition of this response by anti-inflammatory agents may have adverse effect on the lung epithelial repair. In contrast, when this inflammatory response becomes uncontrolled and maladaptive because of too severe or repeated insults, it may become an important mechanism in the development of lung fibrosis.
