# Correction: HMGB1 Accelerates Alveolar Epithelial Repair via an IL-1β- and αvβ6 Integrin-dependent Activation of TGF-β1

**DOI:** 10.1371/annotation/88f820f2-18dd-4d3b-8989-68f170b26b04

**Published:** 2013-10-15

**Authors:** Jean-François Pittet, Hidefumi Koh, Xiaohui Fang, Karen Iles, Sarah Christiaans, Naseem Anjun, Brant M. Wagener, Dae Won Park, Jaroslaw W. Zmijewski, Michael A. Matthay, Jérémie Roux

There is an error in the number of the first author's (JFP) NIH grant. The correct grant number is: GM086416. 

